# Supplementary material for: Growth-Promoting Effects of Ten Soil Bacterial Strains on Maize, Tomato, Cucumber, and Pepper Under Greenhouse Conditions
Source: Plants (Basel). 2025 Jun 18;14(12):1874. doi: 10.3390/plants14121874 (PMC12196750; doi:10.3390/plants14121874)
Supplement: Supplementary file 1 [file plants-14-01874-s001.zip › Figure S1.pdf]

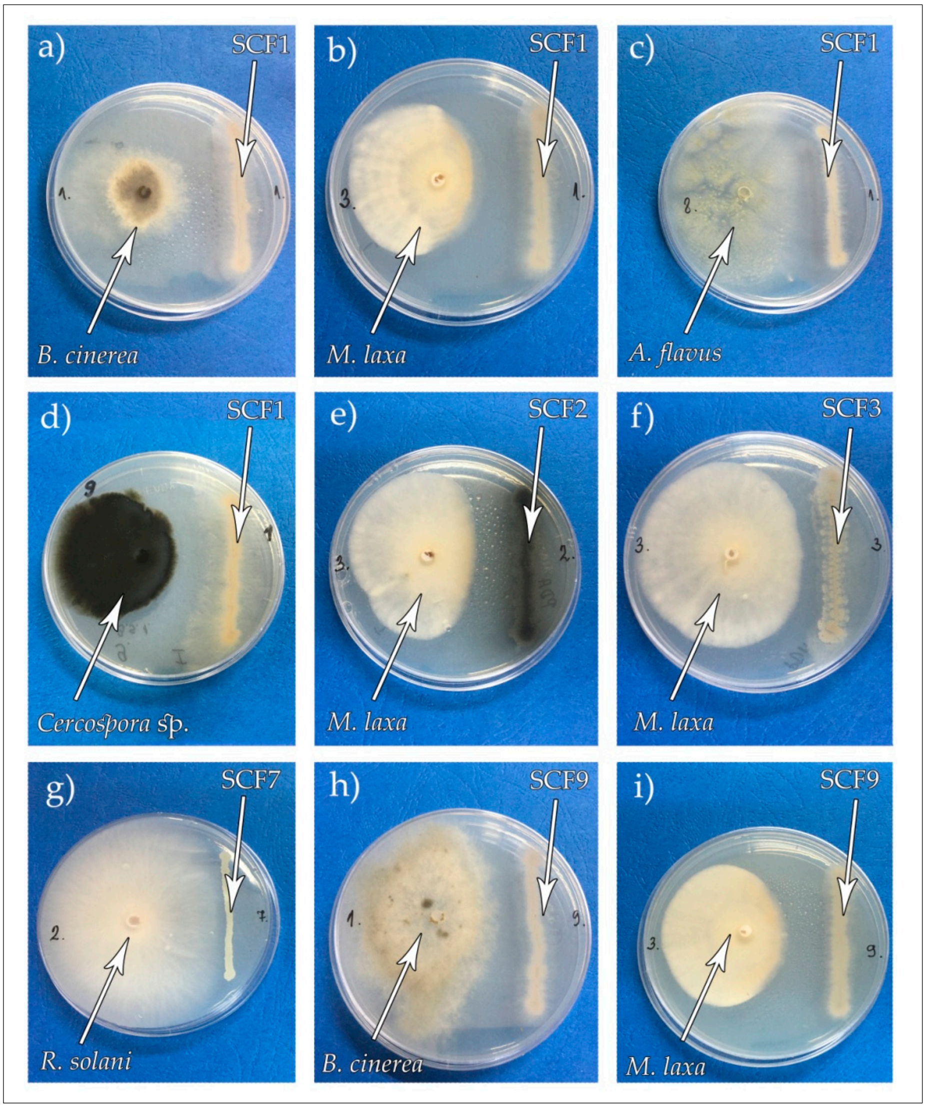

Figure S1. Antifungal activity of bacterial isolates in dual culture assays on PDA plates. Mycelial growth inhibition of: (**a**) *Botrytis cinerea*, (**b**) *Monilinia laxa*, (**c**) *Aspergillus flavus*, and (**d**) *Cercospora* sp. by B. *subtilis* SCF1; (**e**) *Monilinia laxa* inhibition by *B. subtilis* SCF2; (**f**) *Monilinia laxa* inhibition by *B. paralicheniformis* SCF3; (**g**) *Rhizoctonia solani* inhibition by *B. pumilus* SCF7; (**h**) *Botrytis cinerea* and (**i**) *Monilinia laxa* inhibition by *P. putida* SCF9.
