# Supplementary material for: Growth-Promoting Effects of Ten Soil Bacterial Strains on Maize, Tomato, Cucumber, and Pepper Under Greenhouse Conditions
Source: Plants (Basel). 2025 Jun 18;14(12):1874. doi: 10.3390/plants14121874 (PMC12196750; doi:10.3390/plants14121874)
Supplement: Supplementary file 1 [file plants-14-01874-s001.zip › Table S1.pdf]

Table S1. Bacterial isolates from Soil Collection of Fertico (SCF) identified by 16S rRNA sequencing using both 16S ribosomal RNA and core nucleotide databases

Isolates	Databases	Homology to the reference strains	Similarity	Accession Nos.
SCF1	16S ribosomal RNA sequences	*Bacillus halotolerans* DSM 8802	100.00%	NR_115063
	Core nucleotide database	*Bacillus spizizenii* B354/*Bacillus halotolerans* BHFHB3	100.00%	CP118021/OR481937
SCF3	16S ribosomal RNA sequences	*Bacillus haynesii* NRRL B-41327	99.43%	NR_157609
	Core nucleotide database	*Bacillus paralicheniformis* LUB129	100.00%	OR030426
SCF6	16S ribosomal RNA sequences	*Bacillus zhangzhouensis* MCCC 1A08372/*Bacillus safensis* NBRC 100820/*Bacillus pumilus* CIP 52.67	100.00%	NR_148786/NR_113945/NR_115334
	Core nucleotide database	*Bacillus safensis* GOES1/*Bacillus zhangzhouensis* MRC_ER2_58/*Bacillus pumilus* CPO 16.95	100.00%	OL851780/OK605787/OM280134
SCF7	16S ribosomal RNA sequences	*Bacillus zhangzhouensis* MCCC 1A08372/*Bacillus safensis* NBRC 100820/*Bacillus pumilus* CIP 52.67	100.00%	NR_148786/NR_113945/NR_115334
	Core nucleotide database	*Bacillus zhangzhouensis* MCCC 1A08372/*Bacillus safensis* NBRC 100820/*Bacillus pumilus* CIP 52.67	100.00%	NR_148786/NR_113945/NR_115334
SCF9	16S ribosomal RNA sequences	*Pseudomonas oryzihabitans* L-1	99.53%	NR_025881
	Core nucleotide database	*Pseudomonas putida* IHB B 13605	100.00%	KJ767373
