# Supplementary material for: Growth-Promoting Effects of Ten Soil Bacterial Strains on Maize, Tomato, Cucumber, and Pepper Under Greenhouse Conditions
Source: Plants (Basel). 2025 Jun 18;14(12):1874. doi: 10.3390/plants14121874 (PMC12196750; doi:10.3390/plants14121874)
Supplement: Supplementary file 1 [file plants-14-01874-s001.zip › Table S2.pdf]

Table S2. Physiological and biochemical characteristics of SCF isolates.

	**Isolates**									
	SCF1	SCF2	SCF3	SCF4	SCF5	SCF6	SCF7	SCF8	SCF9	SCF10
**Physiological characteristics**										
T (°C)										
4	±	±	±	–	+	±	–	–	±	+
18	+	+	+	+	+	+	+	+	+	+
28	+	+	+	+	+	+	+	+	+	+
40	+	+	+	+	–	+	+	+	+	+
NaCl (%)										
3	+	+	+	–	+	+	+	+	+	+
5	±	±	±	–	±	±	±	±	±	–
7	±	±	±	–	±	±	±	±	±	–
9	–	–	–	–	–	–	–	–	–	–
pH										
4	–	–	–	–	–	–	–	–	–	–
5	+	+	±	±	+	+	+	+	±	+
6	+	+	+	+	+	+	+	+	+	+
7	+	+	+	+	+	+	+	+	+	+
8	+	+	+	+	+	+	+	+	+	+
9	+	+	+	+	+	+	+	+	+	+
**Biochemical characteristics**										
man	3.90	3.54	–	–	–	–	–	4.11	3.49	2.00
EA±SD*	±0.16	±0.19						±0.42	±0.23	±0.00
cel	3.52	2.27	4.02	–	–	2.40	–	2.54	3.38	–
EA±SD*	±0.08	±0.20	±0.31			±0.16		±0.07	±0.23	
lip	1.88	–	1.66	–	–	1.53	–	1.72	2.05	–
EA±SD*	±0.01		±0.05			±0.32		±0.12	±0.08	
am	–	2.08	3.44	–	–	–	–	2.60	2.60	–
EA±SD*		±0.15	±0.48					±0.35	±0.20	
pec	1.55	1.31	2.20	–	–	–	–	1.68	–	–
EA±SD*	±0.18	±0.06	±0.26					±0.05		
cat	+	+	+	–	+	+	+	+	+	+
ur	–	–	+	–	–	–	–	–	–	–
nit	+	–	–	–	–	–	–	–	+	–
cit	–	+	–	–	+	–	+	+	+	+
KA										
l/g	–/–	–/–	–/–	–/–	–/–	–/–	–/–	–/–	–/–	–/+
H_2S	–	–	–	–	–	–	–	–	–	+
gas	–	–	–	–	–	–	–	–	–	+

(–) No growth/ no production; (±) low growth; (+) good growth/ production; man—mannanase; cel—cellulase; lip—lipase; am—amylase; pec—pectinase; EA—enzyme activity; *—values are means ± standard deviations (SD); cat—catalase production; ur—urease activity; nit—nitrate reduction; cit—citrate utilization; KA—Kligler agar: l—lactose and g—glucose fermentation; H_2S—hydrogen sulfide production.
